# ­Exfoliation syndrome and exfoliation glaucoma-associated *LOXL1* variations are not involved in pigment dispersion syndrome and pigmentary glaucoma

**Published:** 2008-07-09

**Authors:** Kollu Nageswara Rao, Robert Ritch, Syril K. Dorairaj, Inderjeet Kaur, Jeffrey M. Liebmann, Ravi Thomas, Subhabrata Chakrabarti

**Affiliations:** 1 Hyderabad Eye Research Foundation, L.V. Prasad Eye Institute, Hyderabad, India; 2 Einhorn Clinical Research Center, New York Eye and Ear Infirmary, New York, NY; 3 Beth Israel Medical Center, New York, NY; 4 Queensland Eye Institute, Brisbane, Australia

## Abstract

**Purpose:**

Single nucleotide polymorphisms (SNPs) in the *LOXL1* gene have been implicated in exfoliation syndrome (XFS) and exfoliation glaucoma (XFG). We have shown that these SNPs are not associated with the primary glaucomas such as primary open-angle (POAG) glaucoma and primary angle-closure glaucoma (PACG). To further establish the specificity of *LOXL1* SNPs for XFS and XFG, we determined whether these SNPs were involved in pigment dispersion syndrome (PDS) and pigmentary glaucoma (PG).

**Methods:**

Three SNPs of *LOXL1* (rs1048661, rs3825942, and rs2165241) were screened in a cohort of 78 unrelated and clinically well characterized glaucoma cases comprising of PG (n=44) and PDS (n=34) patients as well as 108 ethnically matched normal controls of Caucasian origin. The criteria for diagnosis of PDS/PG were Krukenberg spindle, hyperpigmentation of the trabecular meshwork, and wide open angle. Transillumination defects were detected by infrared pupillography, and the presence of a Zentmayer ring was considered as a confirmatory sign. All three SNPs were genotyped in cases and controls by resequencing the genomic region of *LOXL1* harboring these variants and were further confirmed by polymerase chain reaction (PCR)-based restriction digestions. Haplotypes were generated from the genotype data, and the linkage disequilibrium (LD) and haplotype analysis were done with Haploview software that uses the expectation maximization (EM) algorithm.

**Results:**

The *LOXL1* SNPs showed no significant association with PDS or PG. There was no significant difference in the frequencies of the risk alleles of rs1048661 (‘G’ allele; p=0.309), rs3825942 (‘G’ allele’ p=0.461), and rs2165241 (‘T’ allele; p=0.432) between PG/PDS cases and controls. Similarly, there was no involvement of the XFS/XFG-associated haplotypes, ‘G-G’ (p=0.643; [OR=1.08, 95%CI, 0.59–1.97]) and ‘T-G’ (p=0.266; [OR=1.35, 95%CI, 0.70–2.60]), with the PDS/PG phenotypes. The risk haplotype ‘G-G’ was observed in ~55% of the normal controls.

**Conclusions:**

There was no involvement of the *LOXL1* SNPs in patients with PDS and PG. The results further indicate that the associations of these SNPs are specific to XFS/XFG.

## Introduction

Glaucoma is a chronic, progressive neurodegenerative disorder characterized by a specific pattern of optic nerve head and visual field damage, which represents the final common pathway of a heterogeneous group of entities that affect the eye [1,2]. It is the second leading cause of irreversible blindness worldwide, and it has been estimated that it will affect approximately 80 million people by the year 2020 [3].

Exfoliation syndrome (XFS) is an age-related, generalized disorder of the extracellular matrix characterized by the production and progressive accumulation of a fibrillar extracellular material in many ocular tissues and is the most common identifiable cause of open-angle glaucoma worldwide [4]. It plays an etiologic role in open-angle glaucoma, angle-closure glaucoma, cataract, and retinal vein occlusion and has been associated with an increasing number of systemic disorders including vascular disease, hearing loss, and Alzheimer disease [5-8]. Exfoliation syndrome appears to be a disease of elastic tissue microfibrils.

Recently, single nucleotide polymorphisms (SNPs) in the *LOXL1* gene (OMIM 153456) at 15q24.1 have been implicated in exfoliation syndrome and exfoliation glaucoma (XFG) [9]. Two non-synonymous SNPs in exon 1 of *LOXL1* (rs1048661 [R141L] and rs3825942 [G135D]) were demonstrated to exhibit a strong association with XFS and XFG in an Icelandic and Swedish population [9] that was later replicated across multiple populations worldwide [10-19]. It was also shown that *LOXL1* SNPs are not associated with primary glaucomas [20,21].

Pigment dispersion syndrome (PDS; OMIM 600510) and pigmentary glaucoma (PG) are characterized by a disruption of the iris pigment epithelium (IPE) and deposition of the dispersed pigment granules throughout the anterior segment [22]. The classic diagnostic triad consists of corneal pigmentation (Krukenberg spindle); slit-like, radial, mid-peripheral iris transillumination defects; and dense trabecular pigmentation [23]. The iris insertion is typically posterior, and the peripheral iris tends to bow posteriorly [24]. About 80% of patients with PDS are myopes and 20% are emmetropes. The basic abnormality in this hereditary disorder remains unknown.

The frequency with which PDS converts to PG has probably been greatly overestimated. The three studies that have examined patients longitudinally suggest that up to 50% will eventually develop glaucoma [25-27]. However, the true rate of PDS in the general population may be an order of magnitude greater than has previously been suspected [28]. In a retrospective community-based study, 113 patients of whom nine developed PG or elevated intraocular pressure (IOP) that required therapy were newly diagnosed with PDS over 24 years [29]. The probability of converting to PG was 10% at five years and 15% at 15 years.

PDS/PG is an autosomal dominant disorder and was mapped to the 7q35-q36 locus by linkage analysis [30], although the candidate gene is yet to be identified. While POAG shares several clinical features with PDS, there was no evidence of linkage to the POAG-associated 1q21-q31 locus in PDS, indicating that there would be other candidate loci that are yet uncharacterized [31,32].

XFS and PDS are two common disorders that can produce secondary glaucoma through trabecular blockage [22,33]. To further establish the specificity of this association, we studied the involvement of the three XFS- and XFG-associated *LOXL1* SNPs in a cohort of Caucasian PDS and PG patients from New York.

## Methods

### Clinical details of the subjects

The study protocol adhered to the tenets of the Declaration of Helsinki and was approved by the Institutional Review Boards of the New York Eye and Ear Infirmary (NYEE) and the L.V. Prasad Eye Institute. The cohort comprised 78 unrelated patients with PG (n=44) and PDS (n=34) seen at the NYEE between 1998 and 2003 along with 108 normal controls. The diagnoses of PDS/PG were independently confirmed by two surgeons based on the inclusion and exclusion criteria mentioned earlier [33]. The criteria for diagnosis of PDS required the presence of Krukenberg spindles, a deep anterior chamber, wide open angles on gonioscopy, and hyperpigmented trabecular meshwork. Transillumination defects were detected by infrared pupillography. A Zentmayer ring was considered to be confirmatory. A pigment reversal sign was only considered as a soft sign and not categorized as PDS. A diagnosis of PG required PDS plus typical glaucomatous optic disc and visual field damage.

Normal adult individuals without any signs or symptoms of glaucoma and other systemic diseases served as controls. Their visual acuity ranged from 20/20 to 20/40, and their IOP was less than 21 mmHg. The stereodisc exam did not reveal any changes in the optic disc suggestive of glaucoma. All the subjects underwent visual field testing with the Humphreys visual field analyzer (Carl Zeiss Meditec, Dublin, CA). This was essentially a diagnosis of exclusion: normal pattern of neuroretinal rim, absence of notching or thinning of the rim, and disc hemorrhage or nerve fiber layer defects. The cup/disc ratio related to the disc size, the asymmetry of cup to disc ratio less than or equal to 0.2:1 (corrected for size), and the absence of a beta zone peripapillary atrophy were “soft” signs. All the patients and controls were matched with respect to their ethnicity.

### Molecular analysis

**Figure 1:**
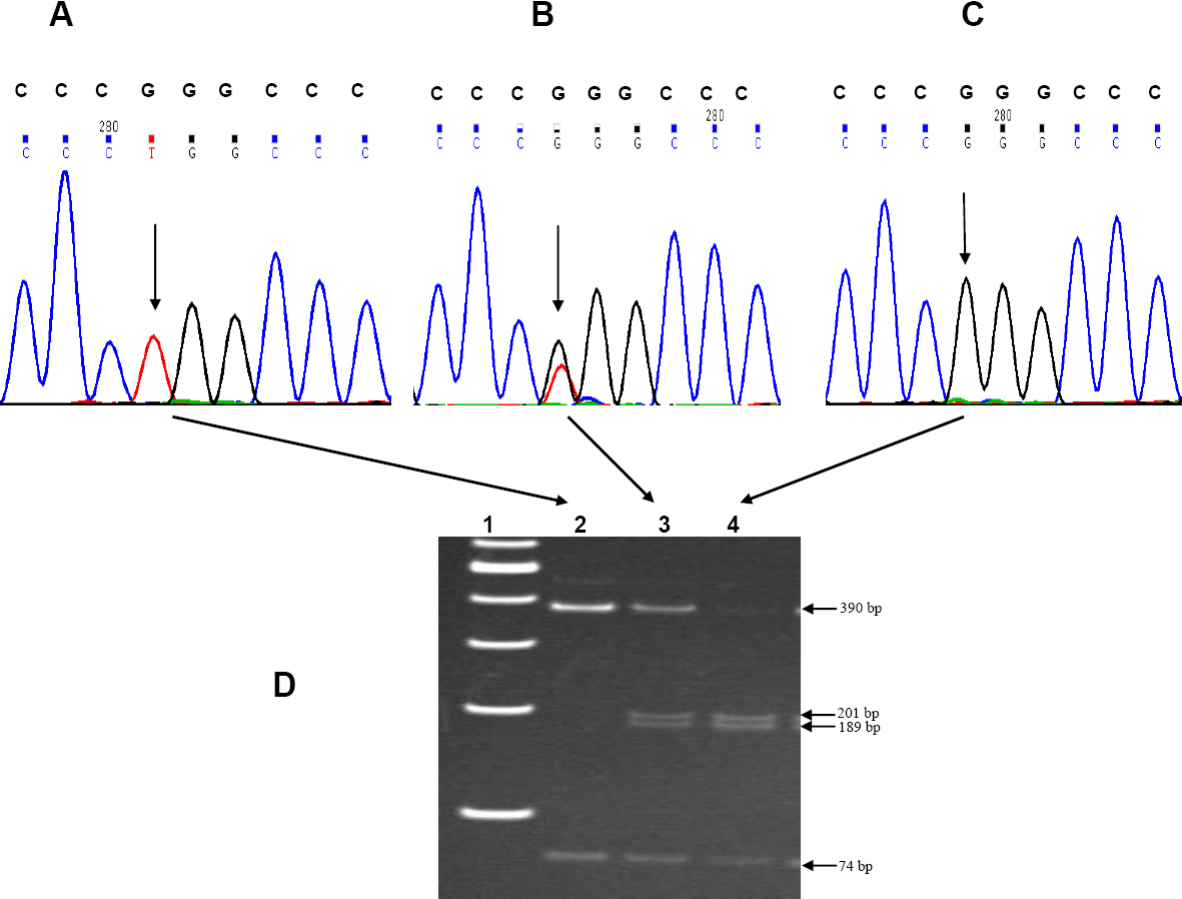
Genotype pattern of the *LOXL1* SNP rs1048661 (R141L). The representative electropherograms show the three genotype patterns for the rs1048661 (G>T) SNP in **A** (TT; homozygous), **B** (GT; heterozygous), and **C** (GG; wild type). The arrow heads indicate the point of substitution. The normal sequence is provided in the upper panel above each electropherogram. **D** demonstrates the confirmation of these variants by PCR-based restriction digestion in a non-denaturing polyacrylamide gel. The PCR amplicon (464 bp) for *LOXL1* (obtained using the primer pairs 5′-GCA GGT GTA CAG CTT GCT CA-3′ and 5′-ACA CGA AAC CCT GGT CGT AG-3′) after digestion with SmaI cleaved into fragments of 201 bp, 189 bp, and 74 bp in the wild type (lane 4). Presence of the variant abolished the site for this restriction enzyme, generating an intact fragment of 390 bp and 74 bp in the individual homozygous for this variant (lane 2). The individual with the heterozygous variant (lane 3) exhibits all the fragments (390 bp, 210 bp, 189 bp, and 74 bp). Lane 1 contains the 100 bp DNA ladder (Gene Ruler^TM^; MBI Fermentas, Vilnius, Lithuania).

**Figure 2:**
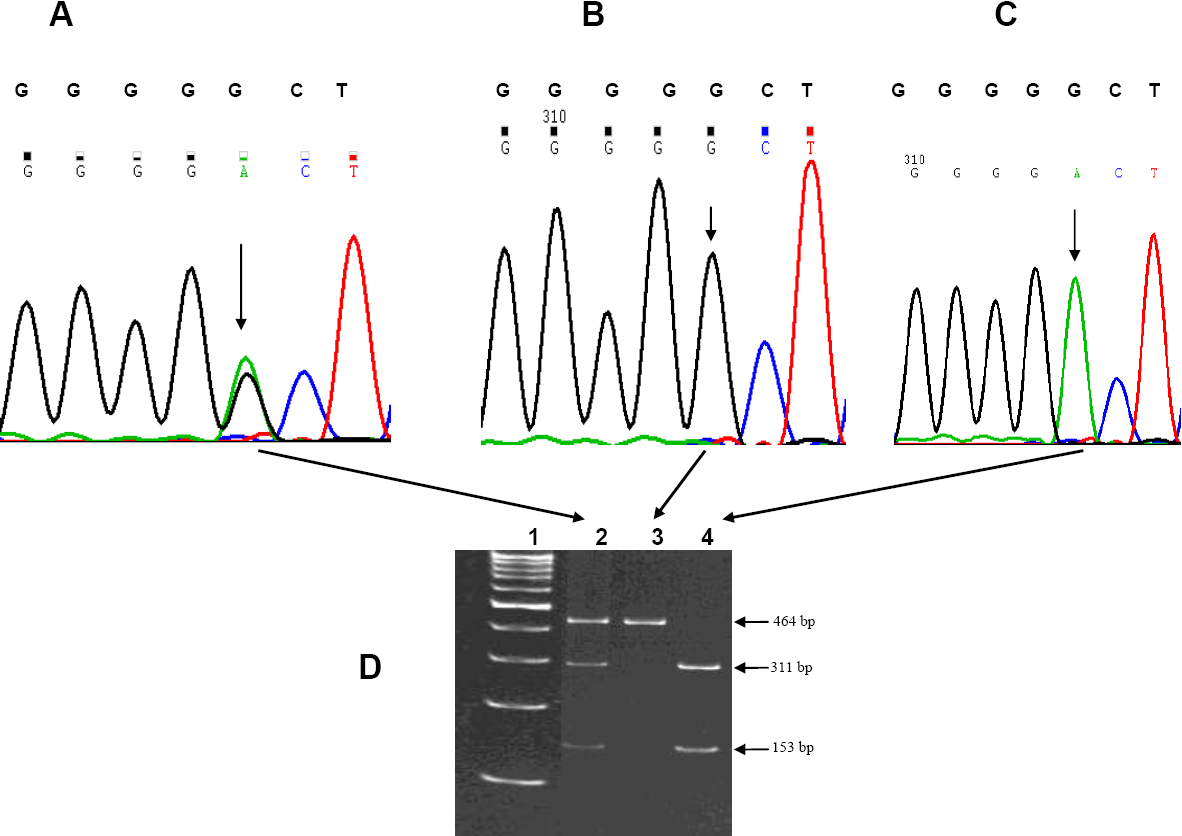
Genotype pattern of the *LOXL1* SNP rs3825942 (G153D). Representative electropherograms show the three genotype patterns for the rs3825942 (G>A) SNP in **A** (GA; heterozygous), **B** (GG; wild type), and **C** (AA; homozygous). The arrow heads indicate the point of substitution. The normal sequence is provided in the upper panel above each electropherogram. **D** demonstrates the confirmation of these variants by PCR-based restriction digestion in a non-denaturing polyacrylamide gel. The PCR amplicon (464 bp) for *LOXL1* (obtained using the primer pairs 5′-GCA GGT GTA CAG CTT GCT CA-3′ and 5′-ACA CGA AAC CCT GGT CGT AG-3′) after digestion with HinfI generated an intact fragment of 464 bp in the wild type (lane 3). Presence of the variation generated a restriction site for this enzyme and cleaved into fragments of 311 bp and 153 bp in the individual homozygous for this change (lane 4). The individual heterozygous for this change (lane 2) exhibited all three fragments (464 bp, 311 bp, and 153 bp). Lane 1 contains the 100 bp DNA ladder (Gene Ruler^TM^; MBI Fermentas, Vilnius, Lithuania).

**Figure 3:**
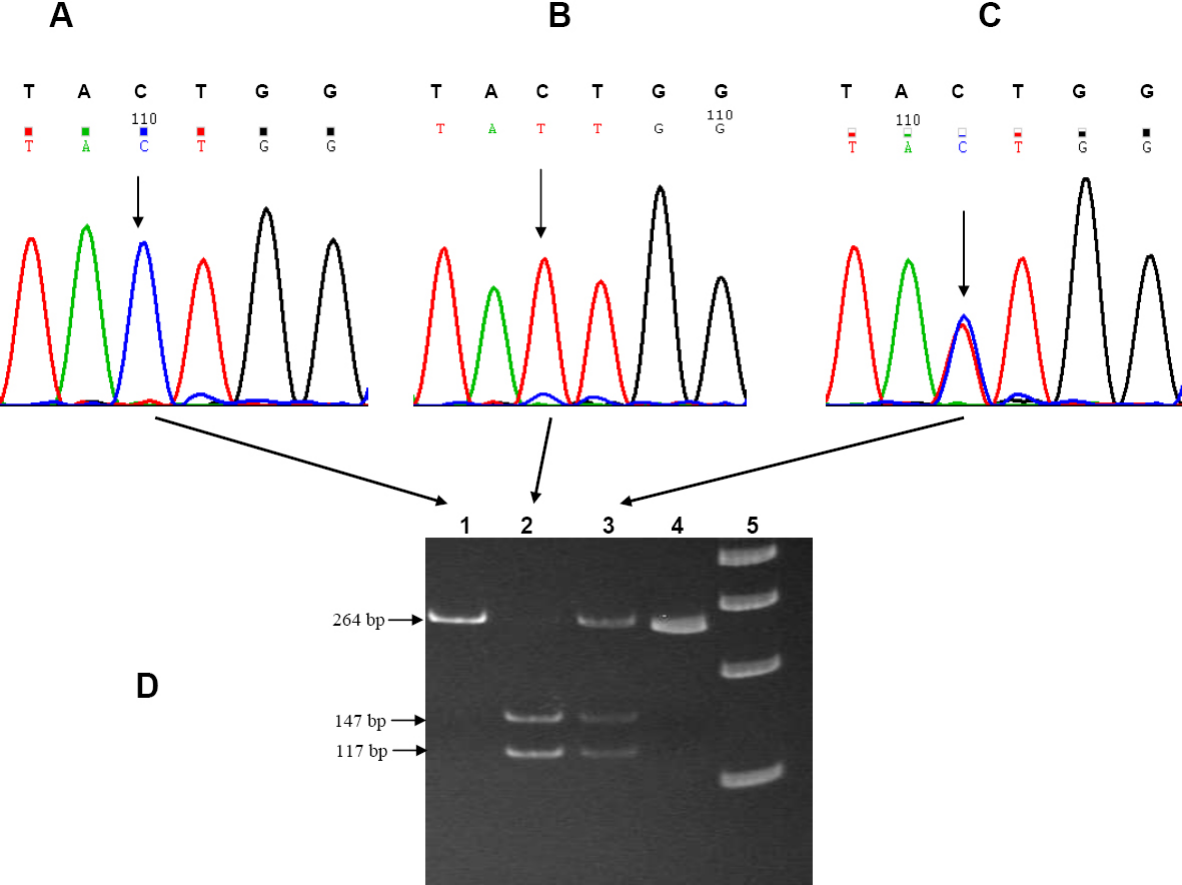
Genotype pattern of the intronic *LOXL1* SNP rs2165241. Representative electropherograms show the three genotype patterns for the rs2165241 (C>T) SNP in **A** (CC; wild type), **B** (TT; homozygous), and **C** (CT; heterozygous). The arrow heads indicate the point of substitution. The normal sequence is provided in the upper panel above each electropherogram. **D** demonstrates the confirmation of these variants by PCR-based restriction digestion in a non-denaturing polyacrylamide gel. The PCR amplicon (264 bp) for *LOXL1* (obtained using the primer pairs 5′-TAG GGC CCC TTG GAG AAT AG-3′ and 5′-GTC CCA TTC CCC TCT CAA TC-3′) after digestion with SspI generated an intact fragment of 264 bp in the wild type (lane 1). Presence of the variation generated a restriction site for this enzyme and cleaved into fragments of 147 bp and 117 bp in the individual homozygous for this change (lane 2). The individual heterozygous for this change (lane 3) exhibited all three fragments (264 bp, 147 bp, and 117 bp). Lane 4 contains an undigested amplicon, and Lane 5 contains the 100 bp DNA ladder (Gene Ruler^TM^; MBI Fermentas, Vilnius, Lithuania).

Peripheral blood samples (5–10 ml) were collected from each subject by venipuncture with prior informed consent, and DNA was extracted by standard protocols [34]. The three SNPs in exon 1 (rs1048661 and rs3825942) and intron 1 (rs2165241) of *LOXL1* were amplified with pre-designed primers; the amplicons were purified and screened by re-sequencing using BigDye chemistry (version 3.1) on an ABI 3100 DNA Analyzer (Applied Biosystems, Foster City, CA) as described earlier [21]. The genotypes of a subset of patients and controls were further confirmed by restriction digestion of the amplicons at 37 °C overnight with appropriate restriction enzymes as detailed earlier [21]. The genotyping was repeated independently by investigators who were masked to the phenotypes. Representative chromatograms displaying all the genotype patterns for these three SNPs are provided in Figure 1 (rs1048661), Figure 2 (rs3825942), and Figure 3 (rs2165241).

### Statistical analysis

The maximum likelihood estimates of allele frequencies, Hardy–Weinberg equilibrium, and haplotype frequencies were estimated from the genotype data at the three SNP loci using Haploview software that uses the expectation maximization (EM) algorithm [35]. Pairwise linkage disequilibrium (LD) between the individual SNPs was calculated using the LD-plot function of this software. χ^2^ analysis was done to assess the significance between the allele frequencies. The odds ratios were calculated to assess the risk of the individual alleles of all three SNPs.

## Results

### Distribution of the *LOXL1* single nucleotide polymorphisms in pigment dispersion syndrome and pigmentary glaucoma

The study cohort conformed to the Hardy–Weinberg equilibrium. The allele frequencies of the three SNPs and their corresponding allele counts are provided in Table 1. There was no significant difference in the frequencies of the XFS/XFG-associated alleles among the PG and PDS patients and controls. The allele frequencies were consistent even after categorizing the data set into PG and PDS phenotypes (Table 1). Similarly, there were no differences in the genotype frequencies of these alleles across these three *LOXL1* SNPs in PG and PDS cohorts (data not shown).

**Table 1:** Allele frequency distributions across PG/PDS cases and controls for the three *LOXL1* SNPs.

**SNPs (Allele)**	**Phenotypes**	**Allele frequency (Counts)**		**p value**
		**Cases**	**Controls**	
rs1048661 (G)	PG+PDS	0.674 (97/47)	0.724 (152/58)	0.309
	PG	0.679 (57/27)	0.734 (152/58)	0.439
	PDS	0.667 (40/20)	0.724 (152/58)	0.389
rs3825942 (G)	PG+PDS	0.852 (121/21)	0.822 (176/38)	0.461
	PG	0.866 (71/11)	0.822 (176/38)	0.368
	PDS	0.833 (50/10)	0.822 (176/38)	0.844
rs2165241 (T)	PG+PDS	0.514 (74/70)	0.471 (99/111)	0.432
	PG	0.524 (44/40)	0.471 (99/111)	0.417
	PDS	0.500 (30/30)	0.471 (99/111)	0.91

### Haplotype analysis of the *LOXL1* single nucleotide polymorphisms

Haplotypes were generated with the three *LOXL1* intragenic SNPs among PG/PDS cases and controls. There was a strong pairwise linkage disequilibrium (LD) between the rs1048661 and rs3825942 (D’=0.89, 95%CI, 0.57–0.97) SNPs and between the rs3825942 and rs2165241 (D’=1.00, 95%CI, 0.81–1.00) SNPs, similar to earlier studies [11-15,17-20].

Four different haplotypes could be generated (with frequency greater than 5%) with these three SNPs in PG/PDS patients and controls. There were no significant differences in the haplotype frequencies between the cases and controls. These results were consistent even after reanalysis of the haplotype data with respect to PG and PDS phenotypes and controls (Table 2).

**Table 2:** Estimated *LOXL1* haplotype frequencies of PG/PDS patients and controls.

**Haplotypes**	**Phenotypes**	**%Cases**	**%Controls**	**p value**
G-G-T	PG+PDS	46.6	45.8	0.881
	PG	48	45.8	0.729
	PDS	44.9	45.9	0.883
T-G-C	PG+PDS	27.9	24.8	0.511
	PG	27.1	24.8	0.683
	PDS	29.1	24.9	0.495
G-A-C	PG+PDS	13.8	16.8	0.451
	PG	12.4	16.7	0.364
	PDS	15.5	16.6	0.831
G-G-C	PG+PDS	7.7	10	0.464
	PG	7.4	10	0.483
	PDS	8.1	9.9	0.662

## Discussion

XFS is the most common identifiable cause of open-angle glaucoma worldwide. It is also associated with extra-ocular abnormalities [36,37]. Recently, intragenic SNPs in *LOXL1* were implicated in XFS and XFG in an Icelandic and Swedish population [9]. Several studies conducted on XFS and XFG worldwide were able to independently replicate these findings in geographically and ethnically diverse cohorts [10-19]. Since *LOXL1* SNPs were implicated in a secondary glaucoma, we analyzed these variations in PDS/PG to establish the uniqueness of this association. To the best of our knowledge, this is the first report to screen for these SNPs in PG/PDS.

The data from the present study show that the three XFS/XFG-associated SNPs were not involved with PG or PDS. The significant associations of the rs1048661 (G allele) and the rs3825942 (G allele) SNPs have been consistent to XFS and XFG across multiple populations worldwide except in Japanese (Table 3). On the contrary, the “T” allele (rs1048661) has exhibited strong association with the Japanese XFS/XFG patients [16,19]. So far, these SNPs have not been involved with primary glaucomas [9,12,20,21]. However, the allele frequencies of the *LOXL1* SNPs in PG/PDS patients were similar to that observed in primary glaucomas (Table 3).

**Table 3:** Worldwide distribution of allele frequencies and their odds ratios for the three *LOXL1* SNPs across all glaucoma phenotypes including the present cohort.

**Type of glaucoma**	**Phenotypes**	**Population [n cases]**	rs1048661 **(G)**			rs3825942 **(G)**			rs2165241 **(T)**			**Reference**
			**Freq**	**OR** **(95% CI)**	**p value**	**Freq**	**OR** **(95%CI)**	**p value**	**Freq**	**OR (95%CI)**	**p value**	
Primary	POAG	Iceland [n=90]	0.711	1.32 (0.96–1.82)	0.085	0.872	1.25 (0.81–1.91)	0.32	0.55	1.36 (1.01–1.83)	0.04	[9]
	POAG	Sweden [n=200]	0.638	0.82 (0.61–1.10)	0.19	0.863	0.87 (0.57–1.31)	0.49	0.488	0.83 (0.63–1.09)	0.18	[9]
	POAG	India [n=112]	0.616	0.70 (0.40–1.24)	0.112	0.83	1.53 (0.78–2.98)	0.105	0.321	0.95 (0.54–1.67)	0.426	[20]
	PACG	India [n=96]	0.667	0.88 (0.49–1.59)	0.332	0.755	0.94 (0.49–1.79)	0.456	0.296	0.82 (0.45–1.50)	0.262	[20]
	POAG	USA [n=331]	0.724	1.02 (0.70–1.51)	0.92	0.771	0.86 (0.57–1.30)	0.54	0.412	0.83 (0.59–1.18)	0.33	[12]
	POAG	Caucasians [n=279]	NA	NA	NA	0.829	NA	0.583	0.424	NA	0.056	[21]
	POAG	African-Americans [n=193]	NA	NA	NA	0.617	NA	0.591	0.237	NA	0.408	[21]
	POAG	Africans [n=170]	NA	NA	NA	0.622	NA	0.217	0.226	NA	0.472	[21]
												
Secondary	XFS	Iceland [n=55]	0.789	2.02 (1.32–3.09)	1.3x10^−3^	0.982	10.1 (4.02–25.36)	8.5x10^−7^	0.74	3.18 (2.12–4.76)	1.9x10^−8^	[9]
	XFG	Iceland [n=75]	0.827	2.56 (1.74–3.77)	1.8x10^−6^	0.987	13.2 (5.59–31.29)	4.1x10^−9^	0.753	3.40 (2.41–4.81)	4.3x10^−12^	[9]
	XFG	Sweden [n=199]	0.834	2.39 (1.72–3.34)	2.7x10^−7^	0.995	27.3 (11.4–65.07)	9.1x10^−14^	0.813	3.78 (2.77–5.14)	3.1x10^−17^	[9]
	XFS	USA [n=72]	0.819	3.03 (1.77–5.17)	0.00003	0.986	9.68 (2.20–42.53)	0.0003	NA	NA	NA	[10]
	XFG	USA [n=50]	0.787	1.86 (1.10–3.15)	0.0222	0.939	3.05 (1.20–7.76)	0.0194	0.667	2.30 (1.40–3.76)	0.001	[11]
	XFS/XFG	USA [n=206]	0.829	1.90 (1.23–2.93)	0.005	0.988	20.9 (8.06–54.39)	1.6x10^−15^	0.76	3.77 (2.56–5.55)	1.2x10^−11^	[12]
	XFS/XFG	American and European [n=287]	0.843	2.26 (1.71–2.99)	7.74x10^−9^	0.959	5.97 (3.77–9.44)	3.1x10^−17^	0.734	2.24 (1.76–2.86)	4.8x10^−24^	[13]
	XFS/XFG	Germany and Italy [n=726]	0.82	2.43 (2.00–2.97)	2.9x10^−19^	0.965	4.87 (3.46–6.85)	8.2x10^−23^	0.765	3.42 (2.85–4.11)	1.9x10^−40^	[14]
	XFG	Europe [n=167]	0.841	2.69 (1.59–4.54)	9.91x10^−19^	0.994	37.29 (6.35–218.02)	5.76x10^−15^	NA	NA	NA	[18]
	XFS/XFG	India [n=50]	0.72	1.49 (0.89–2.51)	0.156	0.92	4.17 (1.89–9.18)	0.0001	NA	NA	NA	[15]
	XFS/XFG	Japan [n=59]	0.008			1			NA	NA	NA	[16]
	XFS/XFG	Japan [n=209]	0.053			0.986	10.87 (4.59–27.25)	1.30x10^−11^	0.017			[19]
	XFS	Australia [n=86]	0.78	1.86 (1.27–2.76)	8.5x10^−4^	0.95	3.81 (1.88–9.02)	7.8x10^−5^	NA	NA	NA	[17]
	PG/PDS	USA [n=78]	0.674	0.79 (0.49–1.25)	0.309	0.852	1.24 (0.69–2.22)	0.461	0.514	1.18 (0.77–1.81)	0.432	Present study

There was no significant association with the *LOXL1* haplotypes either with PG or PDS (Table 2). Since most studies had demonstrated a significant risk with haplotypes generated with the rs1048661 and rs3825942 SNPs [9-15,17-19], a similar exercise was conducted to draw a comparison of the haplotype structure in the present cohort with other studies. The frequency of the risk haplotype with these two SNPs (G-G) was observed in lower frequency among the PG/PDS patients compared to other studies; this risk haplotype was also present in ~55% of the control subjects (Table 4). Unlike previous studies on XFS and XFG, there was no risk associated with the G-G (OR=1.08, 95%CI, 0.59–1.97) and T-G (OR=1.35, 95%CI, 0.70–2.60) haplotypes in PG/PDS (Table 4).

**Table 4:** The estimated haplotype frequencies and their odds ratios based on two SNPs (rs1048661 and rs3825942) across different secondary glaucoma phenotypes in the present cohort and other populations.

**Populations, [n (cases, controls)]**	**Pheno-types**	**G-G haplotype**				**T-G haplotype**				**Reference**
		**% Cases**	**% Controls**	OR (95%CI)*	**p value**	**%Cases**	**%Controls**	OR (95%CI)*	**p value**	
Sweden [399,198]	XFG	83.3	56.1	35.72#	2.2x10^−16^	16.2	31.8	12.36#	1.6x10^−6^	[9]
Iceland [195,14474]	XFG	81.4	49.8	18.94#	3.3x10^−12^	17.3	34.9	5.74#	0.0027	[9]
USA [72,75]	XFS	80.6	48	14.50 (3.27–64.35)	2.7x10^−5^	18.1	40	3.90 (0.84–18.04)	0.12	[10]
American and European [566,658]	XFS	80.2	50.3	4.00 (3.10–5.18)	1.5x10^−7^	15.7	29.6	0.44 (0.33–0.59)	9.0x10^−9^	[13]
Germany and Italy [726,418]	XFS/XFG	78.6	50.7	3.58 (2.98–4.32)	5.2x10^−43^	17.9	34.6	NA	NA	[14]
Europe [167,170]	XFG	83.5	48.8	52.1 (13.85–195.6)	NA	15.9	32.9	14.67 (3.81–56.2)	NA	[18]
India [52,97]	XFS/XFG	64	37	5.74 (2.53–12.98)	9.9x10^−6^	27	36	2.55 (1.07–6.05)	0.129	[15]
Australia [86,2422]	XFS	74	51	2.71 (1.91–3.92)	3.8x10^−9^	22	34	0.54 (0.36–0.78)	7.8x10^−4^	[17]
USA [78, 108]	PG/PDS	52.9	55.3	1.08 (0.59–1.97)	0.643	32.3	26.9	1.35 (0.70–2.60)	0.266	Present study

The asterisk indicates that the odds ratios were calculated with respect to the G-A haplotype. The sharp (hash mark) indicated that the 95%CI were not available in the reported studies.

While PG/PDS occurs relatively early in life, XFS/XFG occurs at a later stage. It has been suggested that certain PDS patients who do not achieve IOP control could later progress to develop XFS/XFG [38]. Based on this, the concept of an “overlap” syndrome has been suggested whereby the sequential appearance of two or more risk factors lead to glaucomatous damage [33].

In summary, we aimed to determine if the *LOXL1* SNPs associated with XFS/XFG were involved in another secondary glaucoma. The high population attributable risks for the high-risk haplotype among the diverse XFS/XFG patients strongly suggest that these variants are exclusive to XFS and XFG [9,13]. The non-association of the *LOXL1* SNPs in our PG/PDS cohort further supports the fact that these are XFS-specific and may not be involved with other secondary glaucomas. Although PG/PDS share certain discrete clinical features with XFS, their underlying molecular mechanisms remain to be elucidated.
